# Programmatic determinants of successful referral to health and social services for orphans and vulnerable children: A longitudinal study in Tanzania

**DOI:** 10.1371/journal.pone.0239163

**Published:** 2020-09-18

**Authors:** Shraddha Bajaria, Ramadhani Abdul, Amon Exavery, Epifania Minja, John Charles, Sally Mtenga, Elizabeth Jere, Eveline Geubbels

**Affiliations:** 1 Health Systems, Impact Evaluation and Policy Department, Ifakara Health Institute, Dar es Salaam, Tanzania; 2 Pact, Dar es Salaam, Tanzania; 1. IRCCS Neuromed 2. Doctors with Africa CUAMM, ITALY

## Abstract

**Background:**

Trained community workers (CWs) successfully deliver health and social services, especially due to greater community acceptance. Orphans and vulnerable children (OVC) and their caregivers (CG) often need support from several sectors. We identified CW, program and referral characteristics that were associated with success of referrals provided to OVC and their CG in Tanzania in a cross-sectoral bi-directional referral system.

**Methods:**

Data for this secondary analysis come from the first two years (2017–2018) of the USAID funded Kizazi Kipya project. Referral success was defined as feedback and service received within 90 days post-referral provision. We analyzed factors that are associated with the referral success of HIV related, education, nutrition, parenting, household economic strengthening, and child protection services among OVC and CG, using generalized estimating equations.

**Results:**

During the study period, 19,502 CWs in 68 councils provided 146,996 referrals to 132,640 beneficiaries. OVC had much lower referral success for HIV related services (48.1%) than CG (81.2%). Adjusted for other covariates, CW age (26–49 versus 18–25 years, for OVC aOR = 0.83, 95%CI (0.78, 0.87) and CW gender (males versus females, for OVC aOR = 1.12, 95%CI (1.08, 1.16); CG aOR = 0.84, 95%CI (0.78, 0.90)) were associated with referral success. CWs who had worked > 1 year in the project (aOR = 1.52, 95%CI 1.46, 1.58) and those with previous work experience as CW (aOR = 1.57, 95%CI (1.42, 1.74) more successfully referred OVC. Referrals provided to OVC for all other services were more successful compared to HIV referrals, with aORs ranging from 2.99 to 7.22. Longer project duration in the district council was associated with increased referral success for OVC (aOR = 1.16 per month 95%CI 1.15,1.17), but decreased for CG (aOR = 0.96, 95%CI 0.94, 0.97). Referral success was higher for OVC and CGs with multiple (versus single) referrals provided within the past 30 days (aOR = 1.28 95%CI 1.21, 1.36) and (aOR = 1.17, 95%CI (1.06, 1.30)) respectively.

**Conclusion:**

CW characteristics, referral type and project maturity had different and often contrasting associations with referral success for OVC versus for CG. These findings could help policymakers decide on the recruitment and allocation of CWs in community based multi-sectoral intervention programs to improve referral successes especially for OVC.

## Background

The needs of orphans and vulnerable children (OVCs) are often not addressed due to lack of integrated care systems, non-supportive structures of society and scarce resources [1–3]. Orphanhood poses a growing public health and social challenge for many communities [4]. In 2015, there were 52 million orphans reported in Africa [5]. While adults in the sub-Saharan African region carry the highest burden of HIV, the consequences affect entire families [4]. The association between orphanhood and HIV has been extensively studied [6, 7]. In 2016, 8% of Tanzanian children under 18 were orphans, many due to losing one or both parents to HIV [8]. Numerous studies [6, 7, 9–11] suggest that orphanhood due to HIV increases children’s vulnerability and risk of adverse health and social outcomes due to the increased infective and social concerns related to HIV. Orphans are at high risk of suffering nutritional deficiencies, lack of access to basic needs, education and health services as well as inadequate care and support [10]. Similarly, vulnerable children living with disabilities, with an ill caregiver (CG), in a child headed household, on the streets or who are victims of abuse also face challenges in accessing essential services and resources for their overall well-being [1, 2]. This consequently necessitates evidence-based programs for supporting and caring for OVCs.

New cadres of community workers (CWs) are increasingly being trained and task shifting is increasingly becoming common [12] for effective scale up of health and social services, especially behavioral interventions [13], including in Tanzania [14]. CWs act as intermediates in linking communities to health, education and social facilities [3, 15], and are efficiently able to provide services and raise awareness due to greater community acceptance [2, 16]. Pre- and in-service training and supportive supervision assist CWs in understanding the program and in orientation on the procedures and standards of the services, positively impacting intervention outcomes [15–18].

Despite training and supervision, not all services can be delivered at community level: functional referral systems that connect patients to health and social facilities are necessary for timely provision of care [16]. Several studies have shown that integrated referrals for interventions for health, economic, nutritional and social development improve health outcomes [19, 20]. Such referrals, whether within one sector or to multiple sectors, can be given using a bi-directional referral system that directs a client to a service that is not offered by the referring provider, after which the receiving service provider feeds back information on the status of service to the referring provider [21].

A five-year (2016–2021) United Stated Agency for International Development (USAID) funded project, Kizazi Kipya (meaning “New Generation” in Swahili), aims to expand access to health, economic and social services among OVCs and their CG in Tanzania. Following identification of eligible beneficiaries, case management based on the needs of all household members is carried out and a care plan for needed services is developed. The CWs act as main liaisons between all actors of the multi-sectoral service provision system, using a national bi-directional OVC referral system.

While numerous studies have revealed the efficiency of CWs in delivering specific interventions such as vaccinations [13], economic strengthening activities and savings schemes [20], food and nutrition services [19], fewer have explored which characteristics of CWs and the programs they work in are associated with successful implementation of the activities in a multi-sectoral project. A previous study from Tanzania showed how the family planning or reproductive services provided by CWs of a community-based project were perceived as inappropriate by community members, depending on age and gender of the CWs [22]. Other studies have also reported the role of CW socio-demographic characteristics such as age, gender or literacy level in service provision and perception of community members [23–25]. This paucity of evidence for a multi-sectoral bidirectional referral system, coupled with a growing demand of CW cadre in delivering community based services, necessitates research to contribute to the little existing knowledge and inform any future program design and implementation that involves CW. We hypothesize that different characteristics of CWs influence referral success differently, more mature programs are associated with higher referral success, as is referral for multiple services, but that referral is equally successful for CG and for OVC, for all service domains. To test this, this study describes the referral cascade for each of the service domains and identifies the characteristics of CWs, program and referral type that are associated with improved success of referrals provided to project beneficiaries.

## Methods

### Study design and setting

This study is a secondary analysis of existing monitoring data from the USAID Kizazi Kipya project. The project aims to scale up access to and provision of essential services to OVC and their CG through collaboration with non-governmental organizations (NGOs), civil society organizations (CSOs), the Government of Tanzania at national, regional and district levels and the communities. Currently in its third year, the project delivers health, economic, education and other social services to OVCs, vulnerable youth and their CG in 152 councils of Tanzania. Community case workers (CCWs) and lead case workers (LCWs) support implementation by identifying service needs of each beneficiary, delivering certain services and providing bi-directional referrals for other services. After beneficiaries met project enrollment criteria and consented to join the project, follow up by CCWs and LCWs was done for health, economic, education and other social services.

### Study site

In its first two years, the project was implemented in 152 councils (out of a total of 185) in Tanzania, i.e. eighty-four scale-up councils where the full intervention was rolled out and sixty-eight sustained councils which only received basic services. Councils were selected into the scale-up group based on their reported high HIV prevalence. For this analysis, data from 68 scale-up councils were included.

### Data quality assessment

Despite the project’s routine data quality assessment, a separate Data Quality Assessment was conducted by Ifakara Health Institute (IHI) in 10% of randomly selected scale-up councils prior to analysis and possible data management and quality issues were identified. Data from 16 councils were then excluded from the analysis due to discrepancies found in the proportion of referral forms given versus the referrals eligible.

### Study population

All CCWs and LCWs (n = 19,502) who worked in the project during the study period constituted the study population. Both CCWs and LCWs provide referrals and deliver monthly services; LCWs have additional responsibilities of supervising, coordinating and managing CCWs, and reporting on the monthly service delivery forms. When the term CW is used in the rest of this paper, it refers to both CCWs and LCWs.

Referrals for OVCs aged 0–19 years and their primary CG provided from January 2017 to September 2018 were included in the analysis, this concerned 146,996 referrals. In the context of this project, the primary CG is a person who has the greatest responsibility for the daily care and rearing of a child [26]. Referrals provided beyond September 2018 were excluded because full 90 days post-referral follow-up data were not yet available at the time of data analysis.

### Data source

Data are routinely collected by the CWs and reported through the project’s standard data collection tools, including: i) a screening and enrollment form that identifies the potential beneficiaries for the project, ii) a family and child asset assessment form that evaluates the household’s needs in order to develop a care plan, iii) a monthly service delivery form that tracks the services provided during monthly household visits, and iv) referral forms which capture type of services beneficiaries were referred to and whether, when and by whom the services were delivered and whether any follow up is needed.

### Details of measurement

We describe the referral cascade as 1) referral provided by community worker, 2) feedback slip received by the community worker for referrals issued, 3) service received as evidenced by the referral feedback slip and 4) feedback and service received within 90 days post-referral.

Characteristics of the community cadre analyzed were age group (18 to 25 years, 26 to 49 years and ≥50 years), gender (male versus female), education level (primary, secondary and post-secondary) and work experience in the project (number of months from CW’s start of work until December 31^st^ 2018, categorized as 0–12 months and more than a year). First date of service provision was substituted if start date of work was missing. Previous work designation was collected during enrollment of the CWs and categorized as follows: CCW/LCW, Community Health Worker (CHW), Home Based Care worker (HBC), Community Facilitator (CF), Empowerment Worker (EW), Para-social worker (PSW), member of a Most Vulnerable Children Committee (MVCC).

District council-specific project maturation (in months) was generated using the date of first referral in the council, up to September 30^th^ 2018. A binary variable for multiple referrals provided to a beneficiary was defined as Yes for a referral given within 30 days of another referral and No otherwise. Multiple related services provided to the beneficiaries were combined to form one service domain (detailed in Table 1). Available CG characteristics such as age (less than 18 years, 18 to 25 years, 26 to 49 years or above 50 years), gender (male versus female) and HIV status (HIV positive, HIV negative or undisclosed HIV status) were included as confounders in the analysis for OVCs as referral success among OVCs may depend on characteristics of their CG.

**Table 1:** Details for each service category.

Intervention	Eligible beneficiaries
**HIV services**	
Referral for HIV counselling services and testing	OVC/ CG
Referral for HIV care and treatment	OVC/ CG
HIV prevention and ART adherence education	OVC/ CG
HIV disclosure support	OVC/ CG
Referral for TB/HIV screening	OVC/ CG
Home based care services	OVC/ CG
Referral for STI treatment services	OVC/ CG
Other HIV related services	OVC/ CG
**Nutrition services**	
Mid-upper arm circumference (MUAC) measurement	OVC
Referral for supplemental feeding services and general food support	OVC/ CG
Nutritional counselling	OVC/ CG
**Educational services**	
Referral for early childhood development	OVC
Referral for life skills education	OVC/ CG
Referral for vocational training	OVC/ CG
Other school services	OVC
**Child protection**	
Referral for birth registration/certificate	OVC
Referral for legal aid and other support	OVC/ CG
Referral for child protection case investigation and response services	OVC
Referral for police services and child/gender desks	OVC
Link to Most Vulnerable Children Committee (MVCC) protection team	OVC
Other general child protection services	OVC
**Parenting education**	
Parenting messages	OVC/ CG
Link to parenting groups	OVC/ CG
**Household Economic Strengthening (HES)**	
Link to Village Savings and Lending Group (VSLG)/ Tanzania Social Action Fund (TASAF)	OVC/ CG
Referral for support for income generating activities	OVC/ CG
Link to agricultural extension support	OVC/ CG

### Data management and analysis

Data analysis was performed using Stata 15 software. Because the Kizazi Kipya project’s key definition of success is increased access to services, referral success was chosen as the main outcome of interest for this analysis. Because individual referrals provide the most fine-grained outcome level possible and therefore allow the most detailed analysis of determinants of success, referrals were chosen as the unit of analysis. Frequencies and proportions were used to describe referral service uptake by OVCs and CGs. A sensitivity analysis was done comparing the districts included into final analysis versus districts excluded; there was no bias.

Since CWs served a specified number of households every month repeatedly, we accounted for the dependent and multi-level nature of the data, with referrals clustered within beneficiaries, beneficiaries clustered within households and households clustered within CWs, creating a multilevel, hierarchical model. To this end, generalized estimating equation (GEE) with logit link function utilizing a binomial distribution family and an exchangeable correlation structure was used to analyze the factors that are associated with referral success of each service type among OVCs and CG. Associations are expressed as Odds Ratios (OR) with 95% confidence intervals; a p-value of 0.05 was considered as cutoff for statistical significance. For the purpose of this analysis, referral success was defined as referral issued by CWs, service received by OVC or CGs and feedback received within 90 days; referrals for which services were given beyond 90 days, not given at all, or no feedback was received were all categorized as unsuccessful referral. The dependent variable was binary; 1 if the referral was successful and 0 for unsuccessful referrals. Independent variables such as CW age, gender, education level, work experience in the project and previous work designation, project components such as type of service of referral, project maturation in the council and whether multiple referrals were given, and CG age, gender and HIV status were included in a univariable model to explore the association with referral success. Factors that were significant in the univariable model were then included in the multivariable analysis.

#### Ethics consideration

Ethics approval was received from the Institutional Review Board (IRB) of Ifakara Health Institute (IHI) (IHI/IRB/No: 001–2017) and the National Institute for Medical Research (NIMR) in Tanzania (NIMR/HQ/R.8a/Vol.IX/3024). Screening and enrollment of beneficiaries into the USAID Kizazi Kipya Project was entirely voluntary and all information is self-reported. All data used for this analysis come from the project’s monitoring database. Data were fully anonymized before the authors accessed and analyzed them.

## Results

### Characteristics of community workers of the project

Table 2 presents the frequencies and proportions for the characteristics of CWs. The study included 19,502 CWs aged 18 or more years. The majority of the CWs were aged between 26–49 years. A higher proportion of the workers were females, slightly more than half had a primary education. Two thirds had been in the project for more than a year. Of all the CWs, 7.2% were LCWs, and the rest were CCWs. Most of the CWs had worked as CCWs or LCWs prior to joining the USAID Kizazi Kipya project and about a quarter had worked as other professionals.

**Table 2:** Characteristics of CWs and program (n = 19,502).

	n	(%)
**Age (years)**		
18–25	3009	15.4
26–49	13656	70.0
50 or above	2837	14.6
**Gender**		
Male	7922	40.6
Female	11580	59.4
**Education level**		
Primary	10296	52.8
Secondary	7976	40.9
Above secondary	1230	6.3
**Work experience**		
Less than a year	6926	35.5
More than a year	12576	64.5
**Community cadre**		
CCW	18098	92.8
LCW	1404	7.2
**Previous designations**		
CCW/LCW	12218	62.7
CHW/HBC/CF	2392	12.3
EW/PSW/MVCC	576	2.9
Others	4316	22.1
**Multiple referrals**	14816	12.6
Project maturation in months Median (IQR)	16	(4, 20)

### Characteristics of caregivers (CG)

Highest proportion of CG were aged between 26 to 49 years (55.8%) and were females (71.2%). Most of the CG were HIV positive (41.1%), whereas 36.8% were HIV negative and 22.1% had not disclosed their HIV status to the USAID Kizazi Kipya project’s CWs.

### Successful referrals for each service type

Table 3 presents the proportion and frequencies of successful referrals for each service type and sub-categories, by beneficiary type. Referrals given to OVC for HIV related services were least successful, especially for HIV counseling and testing services (47.1%) and for Other HIV related services (31.6%). For CGs, referral success for HIV related services was comparatively high, with referrals for STI treatment (79.5%), HIV prevention/ART adherence services (89.2%), HIV care and treatment (87.1%) and HIV disclosure support (85.4%) particularly successful. Referrals given to OVC and CG for nutrition services were successfully completed at the rate of 87.2% and 90.4% respectively, with high success for both referrals for supplemental feeding services / food support and referrals for nutritional counseling. Of the child protection services, for OVC, referrals for linkage to MVCC/child protection teams were most successful (89.3%) and for CG, referrals for child protection case investigation (90.5%). Similarly, high rates of referral success were seen for parenting education. Referrals for HES services were equally successful for CG (88.3%) and OVCs (87.4%), with high completion rates for all referral subtypes.

**Table 3:** Proportion of successful referrals by beneficiary type.

	Overall		OVC		Caregiver	
	n	% (95% CI)	n	% (95% CI)	n	% (95% CI)
**HIV related services**	**41926**	**51.1 (50.8,51.5)**	**31251**	**45.5 (45.1, 45.9)**	**10675**	**80.1 (79.5, 80.8)**
HIV counseling services and testing	29738	52.1 (51.7, 52.5)	22667	47.1 (46.6, 47.5)	7071	79.1(78.3, 79.9)
HIV care and treatment	4578	57.9 (56.8,58.9)	3260	51.3 (50.1, 52.5)	1318	84.9 (83.1. 86.7)
HIV prevention/ ART adherence education	1666	78.3 (76.5, 80.0)	888	73.0 (70.5, 75.4)	778	85.4 (82.9, 87.5)
HIV disclosure support	142	73.6 (66.9, 79.3)	95	71.9 (63.7,78.9)	47	77.1 (64.8, 85.9)
TB/HIV screening	427	53.4 (49.9, 56.8)	318	49.5 (45.7, 53.4)	109	68.9 (61.3, 75.7)
Home based care services	262	86.2 (81.8, 89.6)	149	85.6 (79.6, 90.1)	113	86.9 (79.9, 91.7)
STI treatment services	259	74.2 (69.4, 78.5)	139	70.2 (63.5, 76.2)	120	79.5 (72.3, 85.2)
Other HIV related services	4854	36.7 (35.9, 37.5)	3735	31.6 (30.8, 32.5)	1119	78.8 (76.5, 80.8)
**Nutrition services**	**9179**	**88.5 (87.8, 89.1)**	**5456**	**87.2 (86.3, 87.9)**	**3723**	**90.4 (89.5, 91.3)**
Supplemental feeding services and general food support	3940	89.7 (88.8, 90.6)	2153	88.6 (87.3, 89.8)	1787	91.1 (89.8, 92.3)
Nutritional counselling	5239	87.5 (86.7, 88.3)	3303	86.3 (85.2, 87.3)	1936	89.8 (88.4, 90.9)
**Educational services**	**11707**	**87.6 (86.9, 88.1)**	**9386**	**87.1 (86.4, 87.7)**	**2321**	**89.5 (88.2, 90.6)**
Early childhood development	2074	84.7 (83.2, 86.1)	1615	83.4 (81.7, 84.9)	459	89.8 (86.9, 92.2)
Life skills education	1950	88.0 (86.6, 89.3)	1297	87.2 (85.4, 88.8)	653	89.7 (87.3, 91.7)
Vocational training	737	89.7 (97.4, 91.6)	304	87.6 (83.7, 90.7)	433	91.2 (88.2, 93.4)
Other education services	6946	88.1 (87.4, 88.8)	6170	88.1 (87.3, 88.8)	776	88.2 (85.9, 90.2)
**Child protection**	**4310**	**82.1 (81.0, 83.1)**	**3051**	**81.8 (80.5, 82.9)**	**1259**	**82.9 (80.9, 84.7)**
Birth registration/certificate	1327	78.1 (76.0, 79.9)	938	81.2 (78.9, 83.4)	389	71.4 (67.4, 75.0)
Legal aid and other support	488	82.0 (78.7, 84.9)	379	81.7 (77.9, 84.9)	109	83.2 (75.8, 88.7)
MVCC/Child protection team	86	86.0 (77.7, 91.6)	75	89.3 (80.6, 94.4)	11	68.8 (42.5, 86.8)
Police service / gender desk	74	75.5 (65.9, 83.0)	54	77.1 (65.8, 85.5)	20	71.4 (52.0, 85.2)
Child protection case investigation response	751	89.3 (87.0, 91.2)	541	88.8 (86.1, 91.1)	210	90.5 (86.0, 93.7)
Other general child protection services	1584	82.6 (80.9, 84.3)	1064	78.8 (76.6, 80.9)	520	91.7 (89.1, 93.7)
**Parenting education**	**832**	**89.1 (86.9, 90.9)**	**416**	**87.0 (83.7, 89.8)**	**416**	**91.2 (88.3, 93.5)**
**Household economic strengthening (HES)**	**13603**	**88.2 (87.6, 88.6)**	**2032**	**87.4 (85.9, 88.7)**	**11571**	**88.3 (87.7, 88.8)**
Linked to VSLG/TASAF	8594	88.8 (88.1,89.4)	1376	87.9 (86.2, 89.4)	7218	88.9 (88.2, 89.6)
Supported for income generating activities	3324	87.4 (86.3, 88.4)	368	88.5 (85.0, 91.2)	2956	87.2 (86.1, 88.3)
Linked to agricultural extension support	1685	87.8 (86.2, 89.2)	288	84.9 (80.7, 88.4)	1397	88.4 (86.7, 89.9)

### Bi-directional referral stages

**Fig 1:**
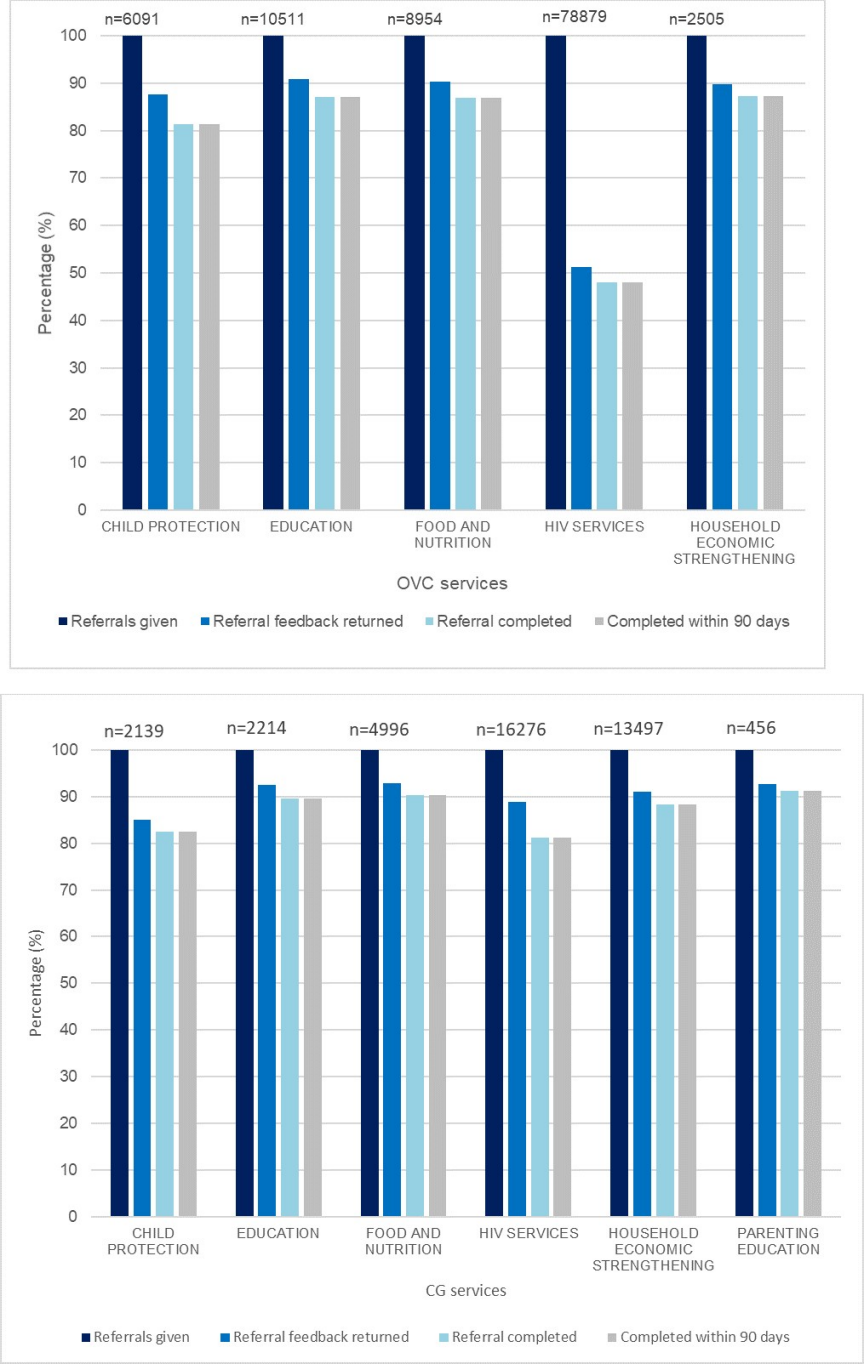
**A.** Percentage of referrals at each stage of bi-directional referral system by services for OVCs. **B.** Percentage of referrals at each stage of bi-directional referral system by services for caregivers.

Bar graphs presented as Fig 1A and 1B present percentages for the three stages of bi-directional referral system of the project; referrals were given, proportion of referral feedback forms returned and proportion of referrals completed, by service type for OVCs and CG respectively. To address the main focus of this analysis, the figures also indicate what proportion of all referrals were completed successfully (within 90 days of provision).

As indicated in Fig 1A, the highest number of referrals for OVCs were provided for HIV related services, followed by education, food and nutrition, child protection and household economic strengthening (HES), in that order. For all types of services, return of the feedback forms ranged from 90% to 51%. The lower proportions of feedback forms returned, out of all referrals given, is a concern for an effective bi-directional referral cascade, especially for HIV services. However, of all the referrals completed, high proportions of referrals were completed successfully within 90 days of provision.

Similar to the referrals pattern for OVC, the highest number of referrals for CG were also provided for HIV related services (Fig 1B), followed by HES services, food and nutrition, education, child protection and parenting education. Similar to OVC, the CG referral cascade had low proportions of feedback forms returned, although the magnitude was smaller; ranging from 92% to 85%. In contrast to OVC, higher proportion of feedback forms for HIV services were returned for CG (89%). Of all the referrals completed, most were completed successfully within 90 days of provision.

### Factors associated with referral success

Table 4 shows the results of univariable and multivariable GEE regression analyses of the factors associated with referral success for OVC and CG.

**Table 4:** GEE logistic regression of factors associated with referral success among OVC and caregivers in Tanzania.

	OVC				Caregiver			
	Univariable (n = 73186)		Multivariable (n = 73186)		Univariable (n = 28872)		Multivariable (n = 28872)	
	OR	95% CI	aOR	95% CI	OR	95% CI	aOR	95% CI
**CW characteristics**								
**Age (years)**								
18–25	1.0		1.0		1.0			-
26–49	0.79***	0.75, 0.83	0.83***	0.78, 0.87	1.03	0.94, 1.15		
Above 50	0.92**	0.87, 0.98	1.01	0.94, 1.08	0.90	0.79, 1.02		
**Gender**								
Female	1.0		1.0		1.0		1.0	
Male	0.95**	0.92, 0.98	1.12***	1.08, 1.16	0.77***	0.71, 0.82	0.84***	0.78, 0.90
**Education level**								
Primary	1.0		1.0		1.0			-
Secondary	1.24***	1.20, 1.28	1.03	0.99, 1.07	0.94	0.88, 1.01		
Above secondary	1.45***	1.33, 1.58	1.03	0.93, 1.14	1.18	0.99, 1.38		
**Work experience**								
Less than a year	1.0		1.0		1.0			-
More than a year	3.65***	3.53, 3.77	1.52***	1.46, 1.58	0.99	0.91, 1.07		
**Previous designations**								
EW/PSW/MVCC	1.0		1.0		1.0		1.0	
CCW/LCW	1.10*	1.01, 1.21	1.57***	1.42, 1.74	1.20	0.99, 1.46	1.23*	1.01, 1.50
CHW/HBC/CF	1.05	0.95, 1.16	1.65***	1.48, 1.84	1.13	0.91, 1.39	1.27*	1.02,1.58
Others	2.15***	1.94, 2.37	2.52***	2.26, 2.81	2.37***	1.91, 2.94	2.24***	1.80, 2.79
**Caregiver characteristics**								
**Age (years)**								
Less than 18	1.0		1.0			-		-
18–25	1.38	0.81, 2.36	1.39	0.76, 2.55				
26–49	1.73*	1.03, 2.93	1.73	0.95, 3.13				
Above 50	1.57	0.93, 2.57	1.57	0.87, 2.84				
**Gender**						-		-
Female	1.0		1.0					
Male	0.74***	0.72, 0.77	0.94**	0.91, 0.98				
**HIV status**								
Negative	1.0		1.0		1.0			-
Positive	1.17***	1.13, 1.22	1.38***	1.32, 1.43	1.08	0.99, 1.17		
Undisclosed	2.05***	1.96, 2.14	1.50***	1.43, 1.58	0.99	0.91, 1.09		
**Project components**								
**Type of service**								
HIV services	1.0		1.0		1.0		1.0	
Nutrition	6.38***	5.89, 6.91	4.12***	3.79, 4.49	2.05***	1.81, 2.32	1.88***	1.66, 2.13
Education	6.65***	6.22, 7.09	5.23***	4.88, 5.61	1.68***	1.44, 1.96	1.63***	1.39, 1.91
Child protection	4.38***	4.01, 4.79	2.96***	2.70, 3.25	0.98	0.84, 1.13	0.93	0.80, 1.09
Parenting education	8.97***	6.43, 12.53	6.71***	4.74, 9.51	2.65***	1.78, 3.93	2.44***	1.63, 3.63
HES	7.96***	6.83, 9.27	7.22***	6.13, 8.50	1.81***	1.67, 1.96	1.72***	1.58, 1.86
**Project maturation (in months)**	1.21***	1.20, 1.22	1.16***	1.15, 1.17	0.96***	0.95, 0.98	0.96***	0.94, 0.97
**Multiple referrals**								
No	1.0		1.0		1.0		1.0	
Yes	1.49***	1.42, 1.57	1.28***	1.21, 1.36	1.14*	1.03, 1.26	1.17**	1.06, 1.30

***p<0.001,

**p<0.01,

*p<0.05

CW characteristics associated with referral success for both OVC and CGs were CW gender and previous work designation. Work experience in the project was only significant for OVC referral success.

CWs aged between 26 and 49 years were slightly less likely than those between 18–25 to provide a successful referral among OVCs (adjusted OR (aOR) = 0.83, 95%CI (0.78, 0.87)). Male CW gender was positively associated with referral success among OVC (aOR = 1.12, 95%CI (1.08, 1.16)) and negatively among CG (aOR = 0.84, 95%CI (0.78, 0.90)). Referrals provided to OVC by CWs with more than one year of work experience in the project were more likely to be successful than if CWs worked for less than a year (aOR = 1.52, 95%CI (1.46, 1.58)). Referral success for OVCs was significantly higher if provided by CWs with previous work designations of CCW/LCW (aOR = 1.57, 95%CI (1.42, 1.74)), CHW/HBC/CF (aOR = 1.65, 95%CI (1.48, 1.84)) or others (aOR = 2.52, 95%CI (2.26. 2.81)) than those who were EW/PSW/MVCC.

The association between service type and referral success seen in Table 3 was even stronger in both the univariable and multivariable GEE analysis, indicating that the clustering by beneficiary, household and district present in the crude data obscured some of the true effect.

For OVC, referrals were significantly more successful for all other types of services (nutrition, education, child protection, parenting education and HES) than for HIV related services, with aORs ranging from 2.99 to 7.22. The duration of project implementation in the district and whether the beneficiary was provided with multiple referrals within the past 30 days were also significantly associated with referral success; for OVC, for each extra month the project had been implemented in the district, the odds of successful referral were 16% higher (aOR = 1.16, 95%CI (1.15, 1.17)); however, for CGs, project maturation was weakly negatively associated with referral success (aOR = 0.96, 95%CI (0.94, 0.97)). Completion for multiple referrals was higher than for single referrals for both OVC (aOR = 1.28, 95%CI 1.21, 1.36) and for CG (aOR = 1.17, 95%CI (1.06, 1.30)).

## Discussion

Referrals provided to OVCs by CWs aged 18–25 years old were more successful than those aged 26–49 years. This could be because younger CWs might be better able to convince OVCs to complete their referrals. This was previously also found in a sexual behavioral change study among adolescents in Tanzania, where peer-counselors close in age to younger beneficiaries but old enough to have authority, successfully counseled clients for behavioral change [27, 28]. Among OVCs, CWs who were aged above 50 years also increased chances of successful referral. This finding is consistent with a study in Kenya [29] showing enhanced performance of older CHWs, possibly due to the cultural emphasis on respecting elders [29, 30]. In contrast, among CGs, CW age had no association with referral success. Male CWs had higher chances of successful referrals among OVCs but lower among CGs, possibly because gender norms in the community view males as being better able to convince others or make decisions, especially for children [31]. However, other studies have shown that among some communities, males have difficulties in passing messages to or convincing women due to cultural norms [32], a possible reason why referrals provided to CG (who were often female) by female CWs were more successful.

Our finding that CW education level was not associated with referral success was in contrast to previous studies that show a significant association between education level of volunteers and their ability of service provision in such programs [24, 33]. While some studies have shown that volunteers with higher education have increased knowledge of the services they provide and the system in general, there is no difference in their ability to convince behavior changes or enable clients [24]. This could also be because education level only takes into account the academic knowledge of CWs, not their understanding of how the project is implemented. As our findings show, for the nature of work of CWs in a project like USAID Kizazi Kipya, increased work experience in the project is more important, as they are trained and supervised specifically to effectively implement project components. Although in univariable analysis, work experience of CWs in the project was strongly associated with the success of referrals given to OVC (OR 3.65), most likely due to better understanding of the project, adjusting for other factors, including project maturation, reduced the effect considerably (OR 1.52). This, and the positive independent effect of project maturation (OR 1.16 per extra month of implementation) indicates that in a complex referral system such as we studied here, the increased acceptance, knowledge and collaboration over time between project actors is at least as important as individual CWs experience. Previous work designation of CWs was also associated with referral success; referrals provided by CWs that had previously worked as CCWs or LCWs were more successful, most likely due to the similar nature of the work and increased understanding of their job’s requirements.

Referral success was high for all services and beneficiaries apart from HIV service referrals to OVC. Previous studies have indicated that while referrals could be advantageous to beneficiaries, barriers such as cost of travel to service providers, other family and work duties, perceived insufficient benefits of the referral or and stigma or refusal to accept their status could hinder referral completion [19, 31]. There could also be barriers from the providers at the service facilities, such as lack of use of referral slips that are essential for following up on referral status for this project, no interest in providing non-clinical health services such as HIV education and counseling [19] or shortage of resources at the facility.

An evaluation of an OVC program of home-visits by trained volunteers in Kenya indicated that one third of the CGs who received health referrals never used that referral and four in ten never used the referral provided to their child, mentioning barriers such as transportation costs, perceived quality of care, attitudes of service providers [3]. Another evaluation of a referral system to access treatment for clients tested HIV positive in Tanzania cited high level of HIV-related stigma, lack of motivation or low readiness to attend clinics due to long distance to the facility, cost of transport, or other household responsibilities as potential explanations for low overall uptake of HIV referrals [31, 34]. The evaluation also reported that referred beneficiaries could be attending HIV clinics without their referral slips, therefore leading to underestimates in proportions of successful referrals [34].

Beneficiaries with more than one referral within the past 30 days had more successful referrals. Although we did not analyze further which combinations of services provided within the past 30 days were the most successful, previous studies have shown that targeting multiple interrelated interventions lead to improved outcomes, especially among people living with HIV [19]. Previous studies have indicated the effectiveness of integrating task shifting by making nurses, other facility staff and CWs in charge of HIV care provision for HIV-infected children, with wider public health measures such as consultations and educational programs [12, 35]. Previous studies have also highlighted the key role of community-based programs in linking clinical and relevant community stakeholders including nutritional, economic strengthening, social development and livelihood services [19, 20, 36].

### Strengths and limitations

A major strength of this study was the large sample size along with use of standardized data collection tools, which reduces the possibility of random error or information bias. The wide geographical coverage ensures that results are applicable to the whole of Tanzania, and may be generalizable to similar contexts in other countries.

This study also had a number of limitations. Referral success of some study clients may have been affected by the combination of distance to the services and seasons, depending on geographical location of the region. For example, during the rainy season, it becomes more difficult for the clients in rural remote areas to timely access the services than for clients in in urban areas. Since it is unlikely though that season is linked to our exposures of interest (CW and program characteristics), we do not expect that seasonality in referral success will have been a source of bias. There may be residual confounding though in our estimate because some potential confounders at program level could not be assessed, such as duration of CWs’ work experience in previous positions, supervision or in-service training received. Additionally, limited data at beneficiary level was available, which precludes a more detailed understanding of the interplay of all factors affecting referral success, such as distance from the facility providing the service, economic status, perceived need for services and perceived stigma associated with seeking help at certain services such as HIV care. Lastly, feedback for HIV services was dependent on how often/whether CWs collect the feedback forms from the health facilities while feedback forms for all other services are given to the beneficiaries, who then give them to the CWs during household visit.

## Conclusion

One of the aims of implementing the USAID Kizazi Kipya, a bi-directional referral program in a highly-burdened population and health system such as that of Tanzania, is to efficiently increase access to services by involving the CW cadre. Our results provide essential evidence on CW and program characteristic that are associated with referral success. If these associations are found to be causal in future studies, characteristics of CWs such as age, gender, previous work experience and education level need to be taken into consideration when selecting CWs for such large-scale programs. The findings also suggest a need of strengthening certain components of referral systems, such as improved process and better follow up of feedback forms, to efficiently implement activities in large multi-sectoral programs. For HIV services for OVC, the links between the CWs, beneficiaries and service providers need to be strengthened in order to have an effective bi-directional system and operational barriers such as loss of feedback forms by the CWs or lack of providers to fill the forms also need to be taken into consideration. Future studies could include qualitative enquiry into the mechanisms underlying successful referrals and analyses using the same monitoring data to explore differences in referral success across more fine-grained geographical areas to explore the influence of contextual variables. These findings will not only aide to improve service provision for the rest of the project period (2019–2021) but can also be used to support future planning of other large-scale programs using a bi-directional referral system in similar settings.

## Supporting information

S1 File Data file for Table 2. (DTA)

S2 File Data file for Table 3. (DTA)

S3 File Data file for Fig 1A and 1B. (DTA)

S4 File Data file for Table 4. (DTA)

## Abbreviations

aOR: Adjusted Odds Ratio
ART: Antiretroviral Therapy
CCW: Community Case Worker
CF: Community Facilitator
CG: Caregiver
CHW: Community Health Worker
CI: Confidence Interval
CSO: Civil Society Organization
CW: Community Worker
EW: Empowerment Workers
GEE: Generalized Estimating Equations
HBC: Home Based Care
HES: Household Economic Strengthening
HIV: Human Immunodeficiency Virus
IHI: Ifakara Health Institute
IQR: Interquartile Range
LCW: Lead Case Worker
MVCC: Most Vulnerable Children Committee
OR: Odds Ratio
OVC: Orphans and Vulnerable Children
PLHIV: People Living with HIV
PSW: Para-social Workers
STI: Sexually Transmitted Infections
TASAF: Tanzania Social Action Fund
USAID: United States Agency for International Development
VSLG: Village Savings and Lending Group
